# Assessment of symmetry and parental satisfaction after use of customized nasal conformers in unilateral cleft lip repair: a randomized controlled clinical trial

**DOI:** 10.1186/s13005-025-00533-6

**Published:** 2025-08-01

**Authors:** Mahmoud Akram Khodir, Saeeda Mahmoud Osman, Hala Ragaa Ragab, Mamdouh Ahmed AboulHassan, Mona Samy Oraby

**Affiliations:** 1 Department of Oral and Maxillofacial Surgery, Faculty of Dentistry, Alexandria University, Champlion Street, El-Azarita, Alexandria, Egypt; 2 Department of General Surgery, Plastic Section, Faculty of Medicine, Cairo University, Al-Saray Street, Al-Manial, Cairo, Egypt

**Keywords:** Nasal conformer, Cleft lip repair, Fisher, Parental satisfaction

## Abstract

**Objectives:**

To evaluate the effectiveness of customized nasal conformers used after unilateral cleft lip repair on nasal symmetry and parental satisfaction.

**Materials and methods:**

Fourteen medically free, non-syndromic children aged 10–24 weeks with unilateral cleft lip were divided into two groups. All patients underwent primary repair using the Fisher technique. Group I received customized nasal conformers fabricated using digital models, while Group II did not. Anthropometric measurements, including nostril height and width, columella deviation angle, and nasolabial angle were used to assess nasal symmetry between both groups. Parental satisfaction was evaluated using the Cleft Evaluation Profile (CEP).

**Results:**

At six months postoperatively, Group I showed no significant difference between cleft and non-cleft sides across all parameters. In contrast, Group II exhibited significant asymmetry in nostril height and width, although no significant difference was observed in the nasolabial angle. CEP scores showed significantly higher satisfaction in fathers for lip, nose, and profile, and in mothers for nose and profile. No significant difference was noted in teeth appearance for either parent group. Intra-examiner reliability was reliable across all parameters.

**Conclusions:**

The use of customized postsurgical nasal conformers in unilateral cleft lip repair was associated with improved symmetry between cleft and non-cleft sides and appeared to reduce relapse, which may have contributed to increased parental satisfaction with the treatment.

**Trial registration:**

Was retrospectively registered at Clinicaltrials.gov on 23/10/2024 (NCT06637488).

**Supplementary Information:**

The online version contains supplementary material available at 10.1186/s13005-025-00533-6.

## Introduction

Cleft lip is among the most common congenital anomalies, ranging from a small indentation up to a complete gap extending to the nose. It can be unilateral or bilateral. It may occur isolated or in conjunction with cleft palate and alveolus [1]. The prevalence of orofacial clefts is approximately 1 in every 1000 live births which can differ according to the type of cleft [2].

Cleft lip can present as part of a syndrome or as a non-syndromic condition [3]. Despite it occurs mainly due to genetic tendency, environmental factors also play a significant role. Environmental factors such as alcohol, drug abuse, smoking, folic acid deficiency and viruses could interfere with fetal development that have serious effect [4].

Several problems can be associated with cleft lip as appearance disturbance of the face, social and psychological challenges, and compromised functions of feeding and speech. Among early complications that could happen following cleft lip repair; complete wound breakdown and upper respiratory tract infection, while vermilion notching and evident scar are common late complications [5].

Scarring and collapsing of nasal cartilage remain the challenge for every surgeon postoperatively [6].

The use of nasal conformers before and after cleft lip surgery is crucial to obtain better treatment results and acquire good symmetry between both sides [7, 8]. Maintaining the shape of the repaired nose after surgery is critical in correction of cleft nasal deformities [9].

The timing of intervention in cleft lip and nose surgery remains controversial. The growth and development of the nose is least affected when the procedure of the primary lip surgery repair is less invasive. Consequently, the presurgical nasoalveolar molding has been used increasingly. However, not all cleft centers are equipped to provide such treatment. The postoperative use of silicone nasal retainers remains popular [10].

There are two available types of nasal stents: ready-made and customized stents based on plaster models. Ready made products are easily obtained but their shapes and sizes are standardized and they can slip from the nasal cavity owing to their poor support [11]. Customized stents based on plaster models that are made postoperatively offer better anatomical fit and support but the process of fabrication is complex, time consuming and with a high cost. These stents cannot be made before the surgery and the key support position cannot be accurately adjusted [12].

Nowadays, the use of nasal conformers that are fabricated after the lip repair surgery are difficult to meet with clinical demands [13]. Thus new techniques of fabrication of customized nasal stents is badly needed. They should have good support and easy to fabricate; 3-dimensional (3D) printing is good choice [14].

Among various materials evaluated, polymethylmethacrylate (PMMA) was found to have appropriate strength, precision and biocompatibility when in contact with the patients [15].

Improving the quality of life of children with cleft lip and overall esthetic and functional satisfaction is the corner stone behind any procedure or modification done by any author. Orofacial conditions are always a distress not only to the patient but the rest of the family as well [16]. Numerous surveys and questionnaires were conducted to assess the impact of different factors on quality of life and overall satisfaction with cleft surgeries outcomes [17].

The aim of the current study was to evaluate the effectiveness of the use of customized nasal conformers after unilateral cleft lip repair on parental satisfaction and to assess symmetry between both sides.

The null hypothesis of the study was that there is no significant difference of using customized nasal conformers on satisfaction of parents to esthetic results.

## Patients and methods

### Study design and setting

This study was a randomized controlled clinical trial carried out between January and September 2024, conducted on children who were admitted to the Department of Oral and Maxillofacial Surgery, Faculty of Dentistry, Alexandria University in Egypt. The present study was approved by the Research Ethics Committee of Alexandria University Faculty of Dentistry (IRB No.001056 - IORG 0008839), as well as full agreement with the declaration of Helsinki [18]. The study was registered in clinicaltrials.gov (NCT06637488) and the design followed the CONSORT 2010 statement for reporting parallel group randomized trials [19].

### Study participants

The present study included children aged 10 to 24 weeks of both genders with unilateral complete cleft lip following rule of 10s for cleft repair [20]. Any syndromic or previously treated patients were excluded from the study.

### Sample size calculation, allocation and randomization

Sample size was estimated based on assuming confidence level = 95% and study power = 80%. The mean columellar deviation was 30.73 ± 7.24 mm for children who received nasoalveolar molding and 49.30 ± 10 mm for the control group [21]. Based on comparison between independent means using the highest SD = 10 to ensure enough study power, a minimum sample size of 6 patients were required per group yielding effect size of 1.857. This was increased to 7 patients to compensate for lost follow up cases. Total sample size = Number per group x Number of groups = 7 × 2 = 14 patients. Sample size was based on Rosner’s method [22] calculated by G*Power 3.1.9.7 [23]. Participants complying with the inclusion criteria were randomly assigned using a computer-generated list of random numbers to one of the two arms. Allocation was performed by a trial independent individual and the allocation ratio is intended to be equal. Allocation was in equal blocks to ensure that the study groups have equal number of participants [24].

### Surgical procedure

#### Preoperative patient preparation

**Fig. 1:**
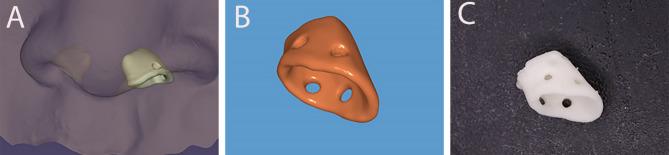
(**A**) Designing the conformer as a mirror image of the normal side (**B**) Virtual design of the conformer (**C**) Real photo showing the nasal conformer after printing

The nasal conformer was prepared for group I via these steps:

- Impressions were taken for the lip and nose using putty addition silicon (Silibest, BMS, Italy) before one week of the surgery.
- Scanning of the silicon impression to get a virtual model of the lip and nose using lab scanner (3 Shape, Copenhagen, Denmark).
- Designing the nasal conformer on 3-Matic software (Materialise, Leuven, Belgium) by obtaining a mirror image of the virtual model and designing the conformer on the borders and inner aspects of the mirror normal side to take the exact anatomical dimensions of the inner nose with 0.7 mm thickness and 0.25 mm larger on outer border of the conformer to overcorrect the nostril dimensions (Fig. 1).
- Preparing two holes with diameter of 1 mm in lateral and medial aspects for the suture material.
- Printing using PMMA material on a 3D printer (Dentcase, Mogassam, USA).
- Disinfection of the conformer using lysoform by immersion 30 min [25].

All Patients underwent laboratory investigations including bleeding profile and haemoglobin level.

Prophylactic antibiotic therapy was administered preoperatively in the form of Cefotaxime (Cefotax, E.I.P.I.C.O, Egypt) **25 mg /kg body weight single dose** intravenously to prevent postoperative infection.

#### Operative procedure

##### I- Group I (Study group) (Figs. 2 and 3)

- The cleft lip and nose was treated using Fisher technique [26].
- Marking was done with methylene blue or surgical markers to preserve anatomic landmarks of the lip and the measurements were made with calipers.
- The incisions and dissection were made following the markings and the repair was done in a three-layer closure: mucosal, muscle, and skin. Interrupted 5 − 0 Vicryl sutures were used for mucosal and muscle layers, and 6 − 0 Prolene interrupted sutures were used for skin.
- Nasal conformer was applied and fixed via 4(0) prolene sutures through 2 holes; medial one interseptal and lateral one to the lateral ala.
- Nasal conformer was removed after 2 months.

**Fig. 2:**
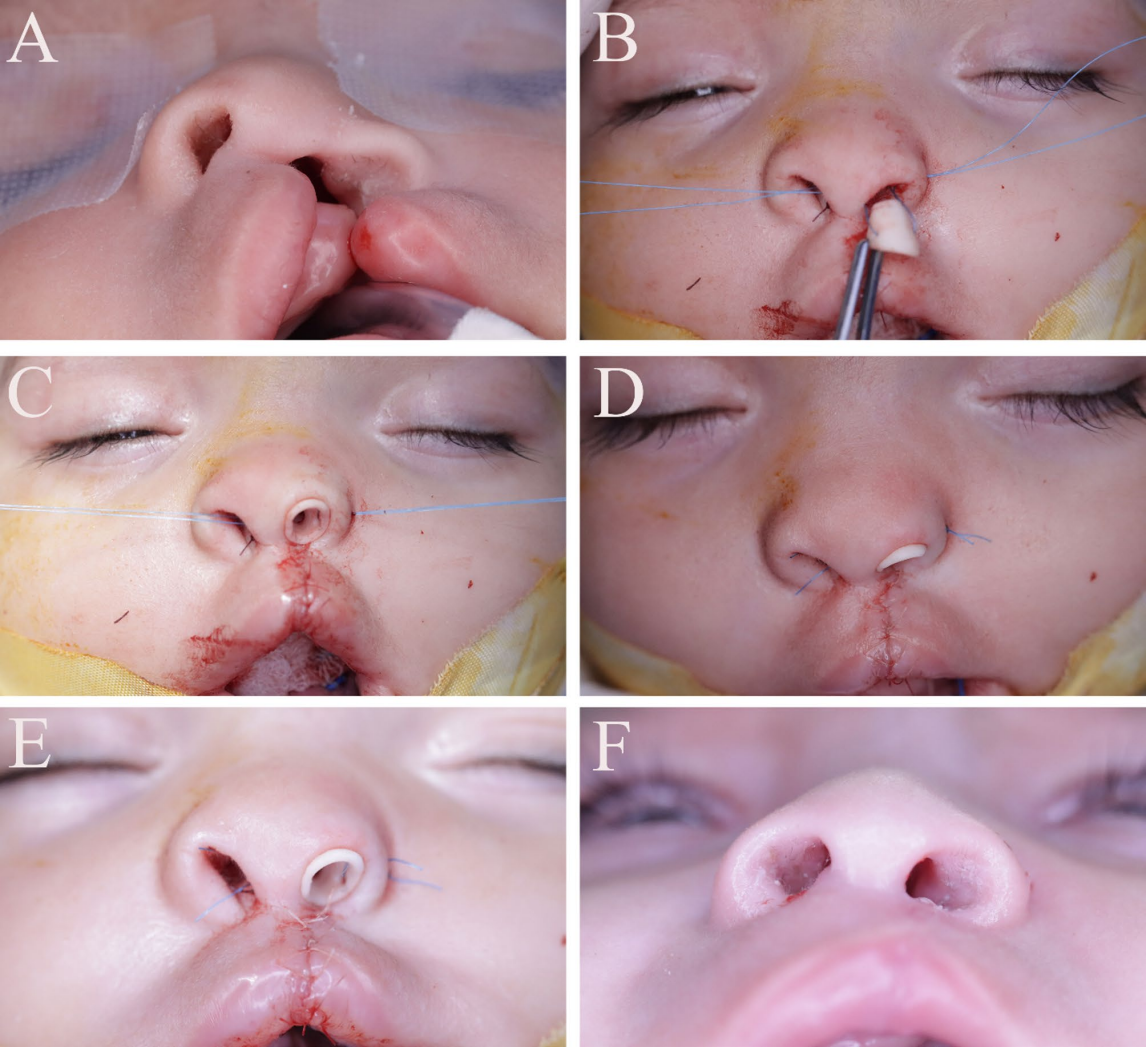
(**A**) Preoperative photo **(B**,** C**) Process of application of conformer by medial and lateral sutures **(D**,** E**) Conformer sutured in place (**F**) Follow-up photo after removal of conformer

**Fig. 3:**
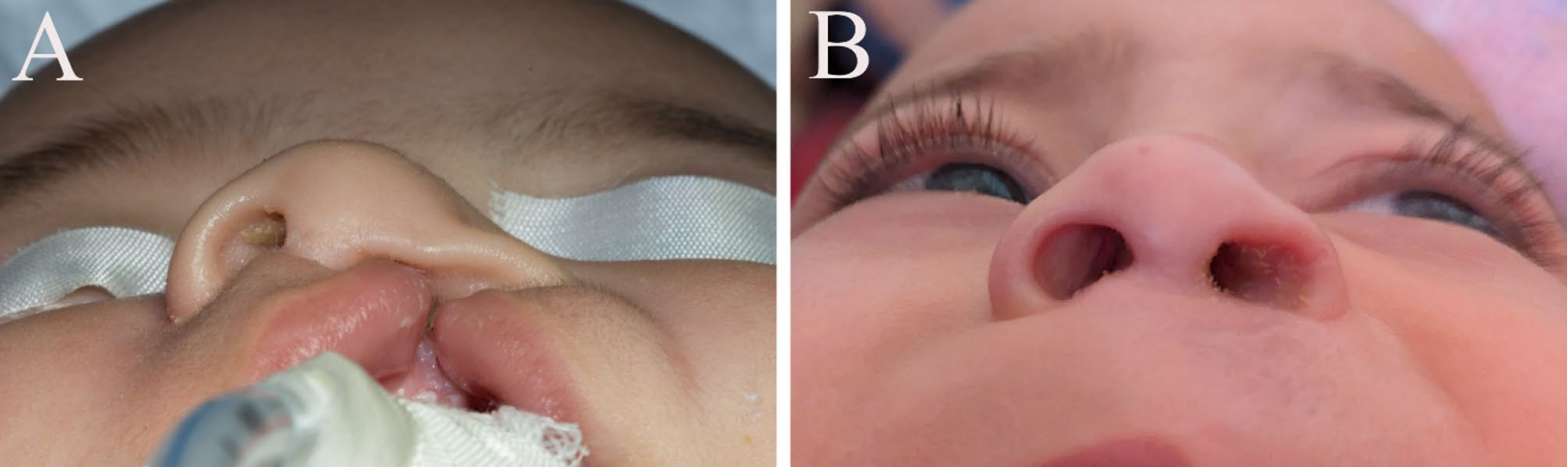
(**A**) preoperative photo (**B**) Six months follow-up showing accepted symmetry

##### II-Group II (Control group) (Fig. 4)

Same technique was done as in group I without the use of the nasal conformer.

**Fig. 4:**
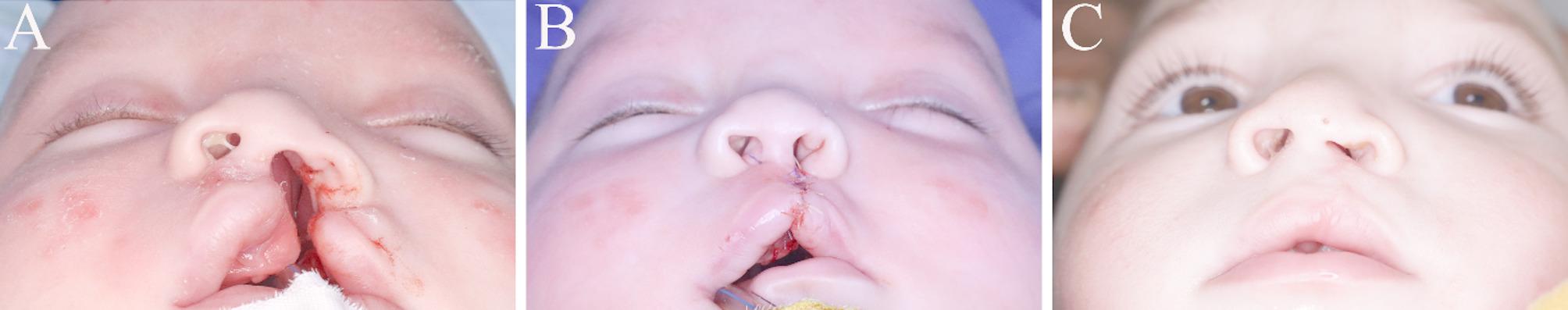
(**A**) preoperative photo (**B**) immediate postoperative (**C**) six months follow-up

#### Postoperative phase

##### Instructions:

-For both groups:

- The parents were instructed to.
  1. Use arm restraints to prevent infants disrupting the repair with fingers.
  2. No breast feeding for the first 2 weeks, feeding only through syringes.

##### Medication

1. Intravenous IM Cefotaxime (Cefotax, E.I.P.I.C.O, Egypt.) 25 mg/kg/12 hours daily for the next 5 days.
2. Gentamicin cream (Garamycin, Memphis, Egypt) 2 cc three times/ day.
3. Paracetamol (Calpol drops, Johnson & Johnson, Ireland) 2.5 cc as needed with maximum 4 times /day.

##### Follow up:

All patients were followed up for 6 months and recalled at the different time points:

T1: Seven days for removal of sutures.

T2: Two months for removal of the nasal conformer for the group I.

T3: Six months for both groups to take final impressions for assessment of different parameters.

### Outcomes variables

#### A-Anthropometric measurements [10]. (Fig. 5) (Table 1)

Measurements were done 6 months postoperative on the digital scans of the final impressions on 3-Matic software (Materialise, Leuven, Belgium).

**Table 1:** Anthropometric measurements

Parameter	Definition
**a-Nostril height**	The greatest vertical distance of the nostril measured in millimeters.
**b-Nostril width**	This measure was defined as the greatest horizontal distance between the inner medial and lateral borders of the nostril aperture measured in millimeters.
**c-Columellar Deviation Angle**	The midline columellar deviation from the vertical reference line was measured.in degrees.
**d-Nasolabial Groove Angle**	The angle between a line tangential to the outer alar basepoint and the horizontal reference line was measured.in degrees.

**Fig. 5:**
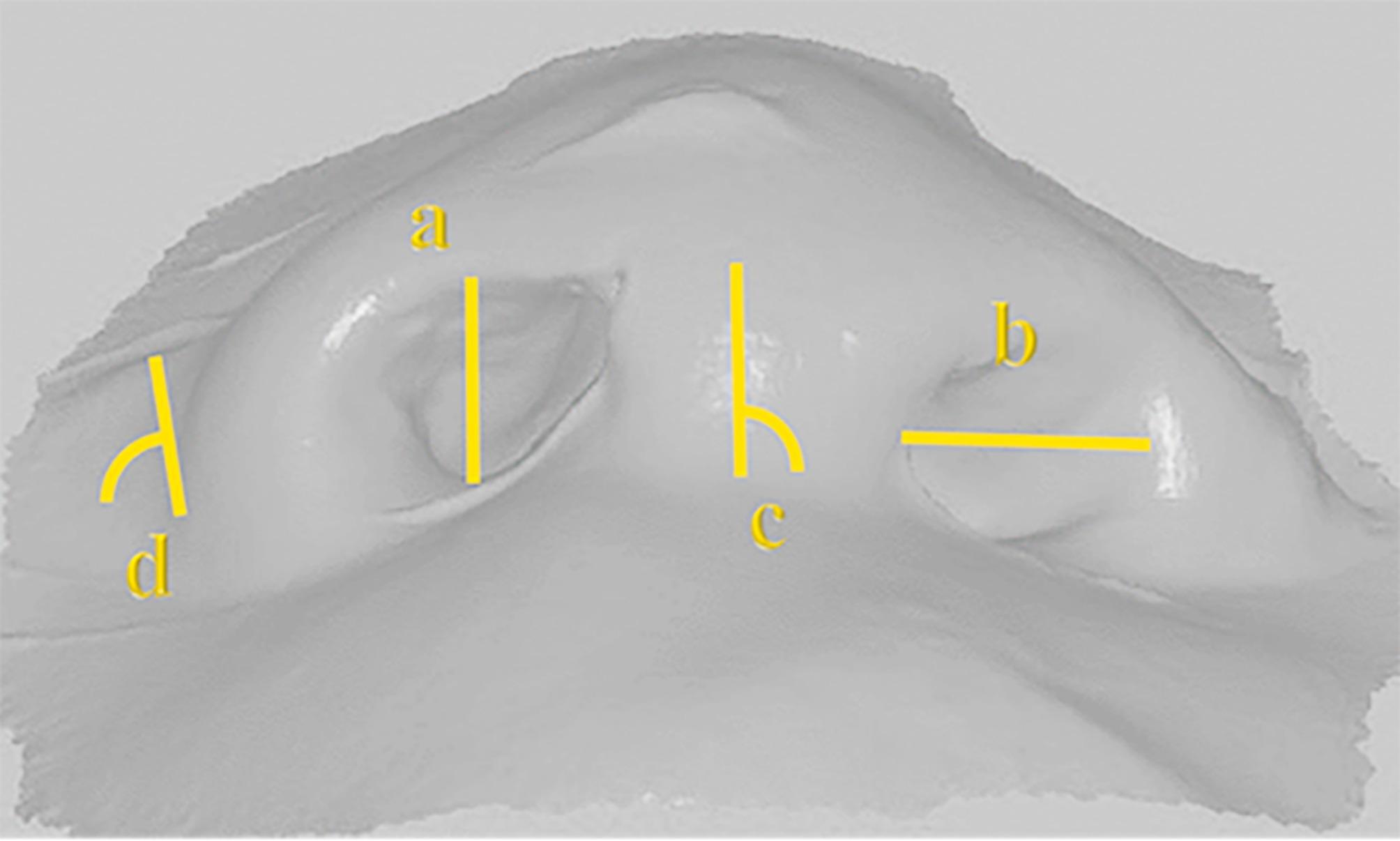
Anthropometric measurements on digital scans (**a**) Nostril height (**b**) Nostril width (**c**) Columellar Deviation Angle (**d**) Nasolabial Groove Angle

#### B-Parental satisfaction

was assessed using simplified version of cleft evaluation profile (CEP) which was first used by Royal college of Surgeons Cleft lip palate audit.This method most commonly employed to evaluate satisfaction with cleft lip and palate surgery outcomes. The simplified CEP was used as the patients were still younger than 1 year so some parameters of the original version could not be applied to this age group. The CEP scale in this study used a 4 Likert scale, that is: Very satisfactory, satisfactory, unsatisfactory and very unsatisfactory. Both fathers and mothers were asked about their satisfaction about 4 areas: appearance of teeth, lips, nose and facial profile [17].

### Statistical analysis

The normality of linear and angular measurements was assessed using the Shapiro-Wilk test and descriptive statistics (Additional File 1). Both types of measurements were confirmed to follow a normal distribution; however, the mean differences between the cleft and non-cleft sides were not normally distributed. Data were summarized using the mean, median, standard deviation (SD), and interquartile range (IQR). Comparisons between cleft and non-cleft sides within each group were performed using the paired t-test, while the Mann-Whitney U test was used to compare deviations in linear and angular measurements, as well as parental satisfaction scores, between the 2 groups. Pearson correlation analysis was conducted to examine relationships between measurements on the cleft and non-cleft sides, and Pearson’s chi-square test was employed to analyze parental satisfaction levels. Intra examiner reliability was assessed using intra class correlation coefficient. All tests were two tailed and the significance level was set at *p* value < 0.05. Data were analyzed using IBM SPSS version 23 for Windows, Armonk, NY, USA.

## Results

**Fig. 6:**
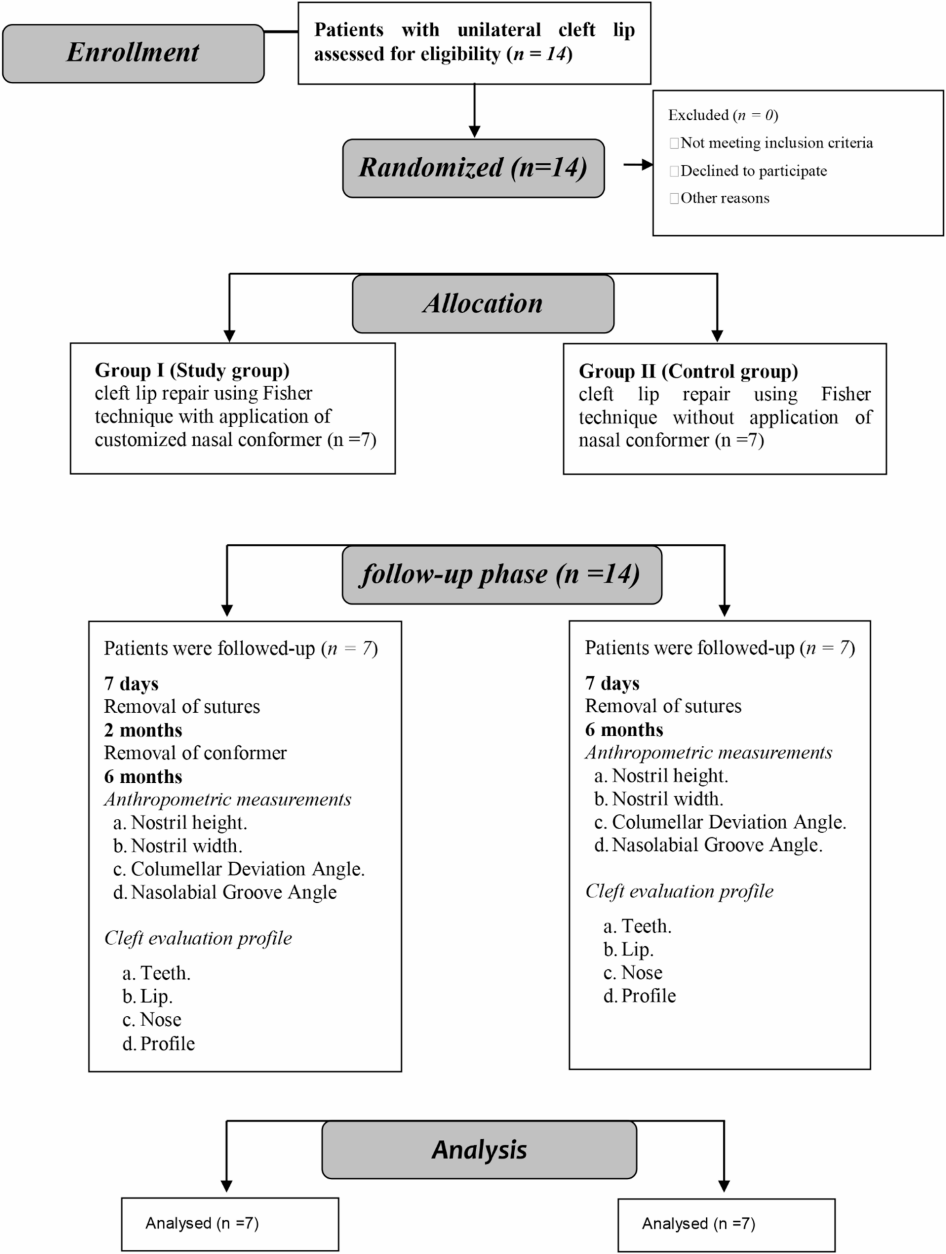
Flow chart of the study showing adherence to CONSORT guidelines

Fourteen patients were included in this study, randomly allocated into 2 equal groups, seven for each, with equal gender distribution. The average age in weeks of group I was 15.57 ± 2.82 and for group II 17.57 ± 3.16 with no significant difference in all demographic data (Table 2). The CONSORT flowchart of the randomized controlled clinical trial was presented in Fig. 6.

**Table 2:** Demographic data of the study sample

Variables	Group I	Group II	*p* value
**Age in weeks (Mean ± SD)**	15.57 ± 2.82	17.57 ± 3.16	0.235
**Gender: n (%)**			
***Males***	3 (42.9%)	4 (57.1%)	1.00
***Females***	4 (57.1%)	3 (42.9%)	
**Cleft side: n (%)**			
***Right***	2 (28.6%)	2 (28.6%)	1.00
***Left***	5 (71.4%)	5 (71.4%)	

Anthropometric measurements within each group between cleft and non-cleft side showed no statistically significant difference within group I for all parameters. Group II showed significant difference for nostril height and width and no significant difference for nasolabial angle (Table 3). Pearson correlation analysis showed statistically significant difference in group I and no statistically significant difference in group II. These results revealed higher symmetry between both sides in group I than group II (Table 4).

**Table 3:** Comparison of linear and angular measurements between both groups

Parameters	Group I				Group II		
	Non cleft side	Cleft side		*p* value^1^	Non cleft side	Cleft side	*p* value^1^
	Mean ± SD				Mean ± SD		
**Nostril height(mm)**	4.89 ± 1.16		4.84 ± 1.16	0.695	6.43 ± 1.30	4.70 ± 1.31	0.035*
**Nostril width(mm)**	8.19 ± 1.48		8.90 ± 1.37	0.082	7.01 ± 1.28	10.83 ± 0.88	< 0.001*
**Nasolabial angle(degrees)**	98.71 ± 6.37		101.43 ± 7.32	0.051	102.14 ± 6.57	111.57 ± 12.79	0.085

*Statistically significant difference at *p* value < 0.05, *p* value^1^: Paired t test

**Table 4:** Correlation of linear and angular measurements between cleft and non-cleft side in both groups

Parameters	Group I		Group II	
	*r*	*p* value	*r*	*p* value
**Nostril height(mm)**	0.97	< 0.001*	0.17	0.723
**Nostril width(mm)**	0.80	0.030*	0.58	0.172
**Nasolabial angle(degrees)**	0.92	0.004*	0.36	0.433

*Statistically significant difference at *p* value < 0.05, r: Pearson correlation coefficient

Comparison of deviation of measurements between both groups showed statistically significant difference in all parameters except for nasolabial angle (Table 5).

**Table 5:** Comparison of deviation of measurements between both groups

Parameters	Group I		Group II		*p* value^1^
	Mean ± SD	Median (IQR)	Mean ± SD	Median (IQR)	
**Nostril height(mm)**	-0.04 ± 0.28	-0.10 (0.20)	-1.73 ± 1.68	-2.20 (2.00)	0.025*
**Nostril width(mm)**	0.71 ± 0.90	0.90 (1.20)	3.81 ± 1.05	3.30 (1.20)	0.002*
**Columella deviation angle(degrees)**	3.14 ± 2.67	2.00 (1.00)	7.29 ± 4.64	7.00 (5.00)	0.081^#^
**Nasolabial angle(degrees)**	2.71 ± 2.95	2.20 (3.70)	9.43 ± 12.12	14.00 (15.00)	0.064

*Statistically significant difference at *p* value < 0.05, *p* value^1^: Mann Whiteny U test, ^#^Independent t test

Parents’ satisfaction levels between both groups were showed in (Table 6) and were analyzed using Pearson’s chi-square test (Table 7). The results showed statistically significant difference between both groups for both fathers and mothers regarding nose and profile appearance. Teeth appearance showed no statistically significant difference. Lip appearance showed statistically significant difference for fathers and no statistically significant difference for mothers.

**Table 6:** Parents’ satisfaction levels between both groups

Parameter	Satisfaction Level	Group I		Group II	
		***Father*** n (%)	***Mother*** n (%)	***Father*** n (%)	***Mother*** n (%)
**Teeth**	***Very unsatisfactory***	0 (0%)	0 (0%)	0 (0%)	0 (0%)
	***Unsatisfactory***	0 (0%)	0 (0%)	0 (0%)	0 (0%)
	***Satisfactory***	4 (57.1%)	6 (85.7%)	6 (85.7%)	7 (100%)
	***Very satisfactory***	3 (42.9%)	1 (14.3%)	1 (14.3%)	0 (0%)
**Lip**	***Very unsatisfactory***	0 (0%)	0 (0%)	0 (0%)	0 (0%)
	***Unsatisfactory***	0 (0%)	0 (0%)	1 (14.3%)	1 (14.3%)
	***Satisfactory***	1 (14.3%)	3 (42.9%)	4 (57.1%)	3 (42.9%)
	***Very satisfactory***	6 (85.7%)	4 (57.1%)	2 (28.6%)	3 (42.9%)
**Nose**	***Very unsatisfactory***	0 (0%)	0 (0%)	1 (14.3%)	1 (14.3%)
	***Unsatisfactory***	0 (0%)	0 (0%)	5 (71.4%)	6 (85.7%)
	***Satisfactory***	2 (28.6%)	3 (42.9%)	1 (14.3%)	0 (0%)
	***Very satisfactory***	5 (71.4%)	4 (57.1%)	0 (0%)	0 (0%)
**Profile**	***Very unsatisfactory***	0 (0%)	0 (0%)	0 (0%)	0 (0%)
	***Unsatisfactory***	0 (0%)	0 (0%)	3 (42.9%)	3 (42.9%)
	***Satisfactory***	1 (14.3%)	2 (28.6%)	3 (42.9%)	3 (42.9%)
	***Very satisfactory***	6 (85.7%)	5 (71.4%)	1 (14.3%)	1 (14.3%)

**Table 7:** Parents’ satisfaction scores between both groups

		Group I		Group II		*p* value	
		*Father*	*Mother*	*Father*	*Mother*	*Father*	*Mother*
**Teeth**	***Mean ± SD***	3.43 ± 0.53	3.14 ± 0.38	3.14 ± 0.38	3.00 ± 0.00	0.254	0.317
	***Median (IQR)***	3.00 (1.00)	3.00 (0.00)	3.00 (0.00)	3.00 (0.00)		
**Lip**	***Mean ± SD***	3.86 ± 0.38	3.57 ± 0.53	3.14 ± 0.69	3.29 ± 0.76	0.035*	0.475
	***Median (IQR)***	4.00 (0.00)	4.00 (1.00)	3.00 (1.00)	3.00 (1.00)		
**Nose**	***Mean ± SD***	3.71 ± 0.49	3.57 ± 0.53	2.00 ± 0.58	1.86 ± 0.38	0.002*	0.001*
	***Median (IQR)***	4.00 (1.00)	4.00 (1.00)	2.00 (0.00)	2.00 (0.00)		
**Profile**	***Mean ± SD***	3.86 ± 0.38	3.71 ± 0.49	2.71 ± 0.76	2.71 ± 0.76	0.008*	0.020*
	***Median (IQR)***	4.00 (0.00)	4.00 (1.00)	3.00 (1.00)	3.00 (1.00)		

*Statistically significant difference at *p* value < 0.05, *p* value: Pearson’s chi-square test

Intra examiner reliability was assessed using intra class correlation coefficient and was shown to be reliable (Table 8).

**Table 8:** Intraexaminer reliability of all measured parameters

Parameters	ICC	95% CI	*p* value
**Nostril height**	0.977	0.892, 0.995	< 0.001*
**Nostril width**	0.970	0.861, 0.994	< 0.001*
**Columella angle**	0.870	0.730, 0.991	0.028*
**Nasolabial angle**	0.992	0.962, 0.998	< 0.001*

*Statistically significant difference at *p* value < 0.05, ICC: Intraclass correlation coefficient

## Discussion

Nasal deformities presented with congenital cleft lip and nose result in a multifaceted structural anomaly that leads to substantial aesthetic and functional challenges. This anomaly impacts every structural layer of the nasal region, including the bone foundation, the inner nasal lining, the cartilage support system, and the outer skin. The severity and manifestation of the deformity are directly influenced by the degree of lip abnormality, ranging from unilateral to bilateral involvement and from minor irregularities to complete structural disruptions [27]. The vast majority of our sample showed unilateral cleft lip of left side, this was in agreement with Begum et al. [28].

Facing these deformities could be achieved by presurgical nasoalveolar molding, surgical primary rhinoplasty and postsurgical application of nasal conformer [29].

Some cleft centers tend to use presurgical nasoalveolar molding as routine and reported their effectiveness in maintaining postoperative nasal contour [30, 31]. However, other reports stated that only 37% of US centers used NAM in their practice [32]. In our protocol we did not rely on the use of NAM owing to its cost and parents’ compliance. Instead, unilateral customized nasal conformers offer a feasible alternative that can be initiated after surgery without interfering with the timing of the primary repair, they were used routinely after primary nasal cartilage dissection, fixed with two sutures in medial and lateral crus for 2 months. This method of fixation solved the problem reported by Tan et al. that the conformer cause discomfort and slippage from the infants [12]. The duration of 2 months aimed to balance between patient comfort and compliance with effectiveness of treatment. Extended use of conformers, while potentially more effective in minimizing nasal relapse, may pose challenges in infants, including irritation, discomfort, and reduced cooperation. Our protocol aligns with findings from a recent systematic review by Nguyen et al. [33], which reported a broad range of stenting durations across studies, including shorter-term protocols similar to ours One solution to overcome this problem was to use larger size of conformer to nostril size or to fix it by tape as documented by Zhang Bin [11]. Although ready-made nasal conformers come with different sizes but nasal cavities are not regular structures, so children may feel discomfort when wearing these devices that can fail to provide proper support in required areas while make unneeded tension in other areas that do not require support [34]. Our design of unilateral type of conformer made it comfortable for the infants and inconspicuous, this is in agreement with Yuzuriha, while he recommended to use bilateral type in cases of columellar tilt [34], our protocol had a solution where it adjusted the columella on the virtual model in the designing phase and the unilateral conformer was fabricated on the corrected columella.

Sykes et al. reported that even after well-executed primary repair, persistent nasal asymmetry is common, especially in the cleft-side nostril, which often appears wider and flatter than the non-cleft side. This structural imbalance can affect both function and esthetics, reinforcing the importance of supportive postoperative devices [27].

The anthropometric measurements after six months showed statistically better symmetry in group I to group II regarding nostril height and width which was in agreement with Al-Qatami et al. [35]. Funayama et al. in 2019 found statistical difference between 2 groups regarding nostril height while non-statistical difference regarding nostril width [10]. Waewsanga in 2021 found no statistical difference in nostril height and width between cleft and non-cleft sides in group I which was in accordance to our results [36]. Columella deviation angle and nasolabial angle showed better results in group I with no statistical difference compared to group II, this was in accordance to Funayama et al. concerning symmetry of nasolabial angle while they found statistical better results regarding columella deviation angle [10].

Parental satisfaction and psychological impact is of great importance, so several studies were conducted to determine satisfaction with cleft surgeries outcomes [17]. Our results showed overall satisfaction for both groups regarding teeth, lips and profile appearance with least levels of satisfaction concerning nose appearance in group II with significant difference to those of group I that showed high level of satisfaction upon the use of nasal conformer for both parents. The use of nasal conformer showed neither complications nor discomfort for the infant and the parents. This was in agreement with the study held by Quentin et al. that used two questionnaires, tolerance and information, to measure quality of life associated with the use of nasal conformers after primary cleft lip repair [37]. A study held by Ha et al. showed lower level of satisfaction with the appearance of nose and teeth [38]. Budihardja et al. reported almost similar results to group II with the mothers least satisfied with the nose appearance [17]. Teeth appearance in our study showed satisfactory levels with no significant difference between two groups. In contrast, studies held in Malaysia and Uganda showed most dissatisfaction with teeth appearance [39, 40]. This may be attributed that our patients were still in their first year of age with no obvious problems appeared in their teeth like malocclusion, caries and crowding, so further follow up is needed when the patients enter mixed and permanent dentition phase. Concerning profile appearance, Fathers in group I showed better satisfaction than group II with significant difference. This was in accordance to Ha et al. and in contrast to a Belgian study that showed lower level of satisfaction with the profile appearance [38, 41]. In our study fathers showed to be more satisfied with the results when compared to mothers as agreed by Ha et al. [38].

Although the study demonstrated promising findings regarding the use of customized nasal conformers, the relatively small sample size is a limitation and further research involving larger, more diverse cohorts is recommended. Another limitation is that this protocol can only serve unilateral cases. Also we recommend long term follow up which is essential to assess the results of the use of the conformer and its impact on the children’s life and future studies comparing different stenting durations would be valuable.

## Conclusion

The use of customized postsurgical nasal conformers in unilateral cleft lip repair was associated with improved symmetry between cleft and non-cleft sides and appeared to reduce relapse, which may have contributed to increased parental satisfaction with the treatment.

## Electronic supplementary material

Below is the link to the electronic supplementary material.

Supplementary Material 1

## Data Availability

No datasets were generated or analysed during the current study.
