# Supplementary material for: Assessment of symmetry and parental satisfaction after use of customized nasal conformers in unilateral cleft lip repair: a randomized controlled clinical trial
Source: Head Face Med. 2025 Aug 1;21:55. doi: 10.1186/s13005-025-00533-6 (PMC12315412; doi:10.1186/s13005-025-00533-6)
Supplement: Supplementary file 1 — Supplementary Material 1 [file 13005_2025_533_MOESM1_ESM.docx]

**Additional file 1**

Results of the Shapiro–Wilk normality test for all linear and angular measurements

| ***Variables*** | *Study* | *Control* | *Decision* |
| --- | --- | --- | --- |
| ***Nostril height(mm)*** | ***0.042**** | *0.056* | *Not normal* |
| ***Nostril width(mm)*** | ***0.036**** | *0.109* | *Not normal* |
| ***Columella deviation angle(degrees)*** | ***0.040**** | *0.406* | *Not normal* |
| ***Nasolabial angle(degrees)*** | ***0.006**** | *0.202* | *Not normal* |
